# One-Step Molten Salt Constructing Double S-Scheme K_0.2_WO_3_/NiO/NiWO_4_ Heterojunction for Photocatalytic CO_2_ Reduction

**DOI:** 10.3390/molecules30081804

**Published:** 2025-04-17

**Authors:** Wentao Xiang, Zhenzhen Yu, Renwu Gao, Zhichao Yi, Kun Gong, Kangqiang Lu, Weiya Huang, Changlin Yu, Zeshu Zhang, Man Zhou, Kai Yang

**Affiliations:** 1 School of Chemistry and Chemical Engineering, Jiangxi University of Science and Technology, Ganzhou 341000, China; 2 School of Chemical Engineering, Guangdong University of Petrochemical Technology, Maoming 525000, China; 3 Ganjiang Innovation Academy, Chinese Academy of Sciences, Ganzhou 341000, China; 4 School of Rare Earths, University of Science and Technology of China, Hefei 230041, China; 5 School of Pharmaceutical Sciences, Gannan Medical University, Ganzhou 341000, China

**Keywords:** NiO, K_0.2_WO_3_, NiWO_4_, S-scheme heterojunction, photocatalysis, reduction of CO_2_

## Abstract

Rapid charge separation and transfer is the key scientific problem in photocatalysis. The construction of S-scheme heterojunction is one of the effective strategies to promote charge separation and maintain the strong redox properties. Herein, the NiO, K_0.2_WO_3_, and NiWO_4_ ternary double S-scheme K_0.2_WO_3_/NiO/NiWO_4_ heterojunction (W/NiO) was created by a one-step molten salt method. Ultraviolet-visible (UV-Vis) diffuse reflectance spectra, photoluminescence (PL) spectra, photoelectrochemistry tests, and other analyses revealed that the double S-scheme heterostructure broadened the spectral response range of NiO and promoted its separation of photocarriers. Compared with pristine NiO, the modified double S-scheme heterojunction enhanced the surface adsorption of water molecules and the accumulation of intermediate product of HCOO^−^, and optimized the CO_2_ reduction system, realizing the improved CO yield of 373 μmol·g^−1^·h^−1^ in Ru(byp)_3_^2+^/ethanolamine of CO_2_ reduction system. This study indicates that double S-scheme heterojunction could facilitate efficient photogenerated charge transfer and separation, thereby achieving high activity and selectivity for CO_2_ photoreduction. Our work provides a reference for the one-step construction of double S-scheme heterojunction.

## 1. Introduction

Energy shortage and environmental pollution are the most serious problems faced by human social development. At present, the accelerating consumption of fossil energy in the world has led to increased greenhouse gas emissions, mainly CO_2_, in the atmosphere, breaking the carbon balance of nature [1]. Since the late 19th century, the atmospheric concentration of CO_2_ has increased from 280 ppm to the current 400 ppm. In this context, exploring effective techniques to reduce CO_2_ concentration in the atmosphere has become the key research direction of various governments and scientists. Among several feasible strategies, photocatalytic CO_2_ reduction is particularly attractive, which can be performed at room temperature and constant pressure. The required energy can be directly provided by renewable solar, truly realizing the recycling of carbon elements [1,2,3].

Photocatalysis of CO_2_ reduction involves a process where the excited electrons on the conduction band bind and react with the adsorbed CO_2_ and protons under light irradiation [4,5,6]. The electron migration and CO_2_ activation processes of the photocatalytic process are essentially an energy conversion process. However, charge separation plays the key role in achieving high activity, that is, it is crucial to inhibit the recombination of photogenerated charges by an appropriate co-catalyst over the bulk semiconductor to achieve efficient photocatalytic performance.

As a traditional wide-gap semiconductor, NiO has a wide application prospect in the nanoscale due to excellent conductivity, good chemical stability, and non-toxicity [7]. NiO is also a commonly used low-cost, low-pollution photocatalyst with a band gap of about 3.6–4.0 eV [8]. The wide band gap and high recombination of photogenerated carriers limit the separate application of NiO in photocatalysis. Therefore, the design and synthesis of novel NiO-based photocatalyst that can effectively utilize and convert solar energy is the most urgent and serious challenge.

In the design of a semiconductor in photocatalysis, lots of means were often adopted to modify the semiconductor for achieving efficient charge separation, for instance, the construction of heterojunction is a common method [9,10]. Heterostructure is the combination of two or more kinds of semiconductors [11,12] and it exhibits the following advantages: (i) oxidation and reduction reactions can occur in different semiconductors, inhibiting the reverse reactions; and (ii) heterogeneous interface facilitates charge separation. In recent years, many types of heterojunction semiconductor heterojunctions have been developed [13,14,15]. For example, the S-scheme heterostructure can retain the optimal conduction band position and the most positive oxidation position, which is similar to the process of photosynthesis in nature [16,17]. Yang et al. [18] prepared ZnIn_2_S_4_-NiO/BiVO_4_ heterojunction, effectively promoting the separation of photogenerated carriers and exhibiting the enhanced photocatalytic hydrogen production and simultaneous degradation of HCHO performances. It is urgent to develop NiO-based S-Scheme heterojunction to achieve efficient charge separation.

Herein, ternary a K_0.2_WO_3_/NiO/NiWO_4_ heterojunction was constructed by a one-step molten salt method. The component and physical/chemical properties of the developed materials were investigated by a series of characterizations. The activity test of CO_2_ photoreduction for prepared materials was evaluated in a heterogeneous catalytic system of ruthenium Ru(bpy)_3_^2+^/ethanolamine. The photocatalytic mechanism over K_0.2_WO_3_/NiO/NiWO_4_ was proposed based on experimental evidence.

## 2. Results and Discussion

### 2.1. Structures and Properties of Photocatalysts

**Scheme 1:**
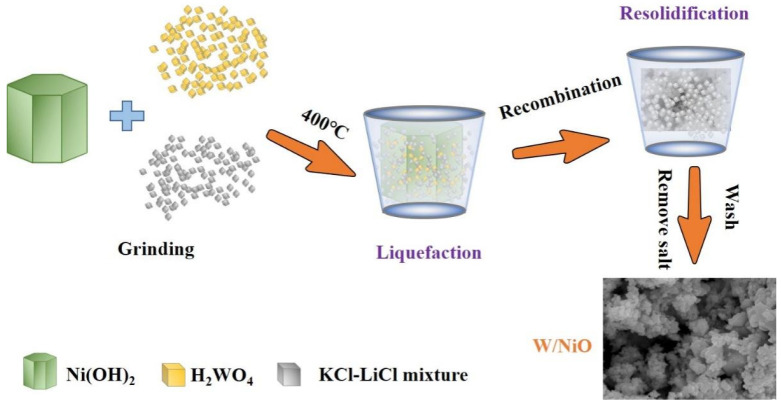
Diagram describing synthesis of W/NiO photocatalyst.

**Figure 1:**
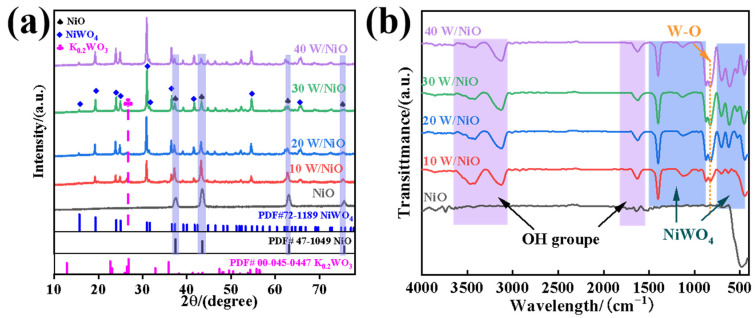
XRD patterns (**a**) and FT-IR diagrams (**b**) of NiO and mW/NiO.

Scheme 1 shows the schematic diagram of W/NiO synthesis. Firstly, Ni(OH)_2_ was synthesized by a precipitation method. Secondly, the mixture of Ni(OH)_2_, commercialized tungsten acid, and KCl-LiCl was introduced into a crucible and treated under 400 °C. The precursors could be decomposed and recomposed into a heterogeneous structure. As presented in the XRD patterns of the typical samples of Figure 1a, pristine NiO was identified to green nickel NiO (JCPDS PDF#47-1049). The characteristic diffraction peaks of NiWO_4_ were generated in the reaction system with the addition of tungstic acid, where 2θ = 15.6°, 19.3°, 24.0°, 25.0°, 30.9°, 36.6°, 41.7°, and 54.7° corresponding to (010), (100), (011), (110), (111), (002), (102), and (221) crystal planes of NiWO_4_ (JCPDS PDF#72-1189), respectively [19,20]. With the increase in the amount of tungstic acid, the intensities of the diffraction peaks of NiWO_4_ and NiO reinforce and weaken, respectively, indicating that NiWO_4_ is more easily generated with the accumulation of W/Ni ratio. Interestingly, the characteristic diffraction peak of K_0.2_WO_3_ (JCPDS PDF#00-045-0447) also appears at 2θ = 27.8°, corresponding to the (200) crystal plane. In addition, with the addition of tungstic acid, the diffraction peaks of K_0.2_WO_3_ gradually weaken and they eventually disappear at the W/Ni ratio of 0.4. The above results indicate that tungstate will preferentially form potassium-tungsten bronze K_0.2_WO_3_ in a K^+^-rich high-temperature environment at a low W/Ni ratio, and the part of tungstate will combine with NiO to form NiWO_4_. Once the ratio of W/Ni increases, WO_3_ generated by the decomposition of tungstate is more likely to combine with NiO to form NiWO_4_, contributing to the weakened diffraction peaks of K_0.2_WO_3_.

To further study the phase composition and surface state of the prepared samples, the typical NiO and mW/NiO samples were tested by FT-IR. As shown in Figure 1b, the stretching characteristic peaks around 480 cm^−1^ correspond to the Ni-O bond [21]. Compared with NiO, mW/NiO heterojunctions exhibit the additional W-O and W-O-W stretching vibration peaks belonging to NiWO_4_ at ranges of 450–750 cm^−1^ and 850–1475 cm^−1^, respectively [22]. In addition, the stretching vibration at around 825 cm^−1^ is identified as the W-O bond. The above results indicate the successful architecture of ternary heterojunction phases consisting of NiO, NiWO_4_, and K_0.2_WO_3_. In addition, the characteristic peaks near 1631, 3141, and 3440 cm^−1^ correspond to the surface absorbed hydroxyl [23,24], and mW/NiO heterojunctions exhibit significantly stronger adsorption of hydroxyl, which proves that protonation of CO_2_ is easier to proceed over mW/NiO.

**Figure 2:**
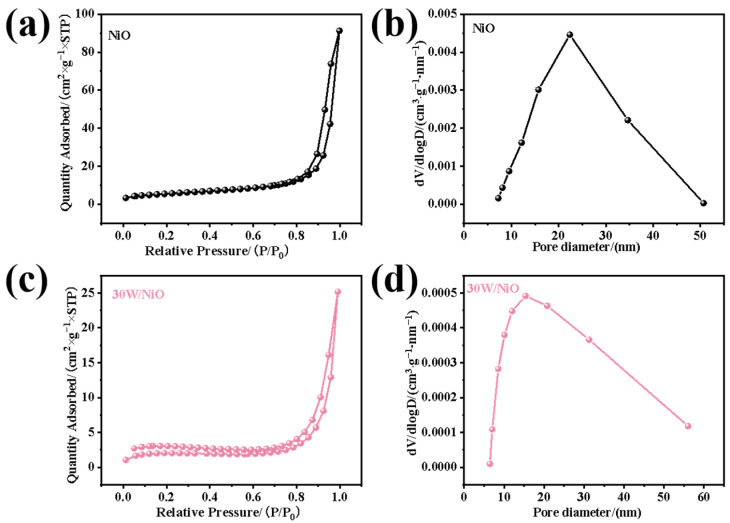
(**a**,**c**) N_2_ adsorption/desorption curves and (**b**,**d**) pore size distributions of NiO and 30W/NiO.

N_2_ adsorption/desorption measurements were utilized to explore the specific surface areas and pore size distributions. As shown in Figure 2a,c and Figure S1, according to Brunel classification, N_2_ adsorption–desorption isotherms for NiO and mW/NiO belong to type IV [25], while there is obvious lag between adsorption and desorption, indicating the presence of slit holes. Compared with pristine NiO, the adsorption–desorption curves of mW/NiO are gradually shifted downward with the increase in W/Ni ratio according to Figure S1 and Table S1, which may be due to the blocked channel of NiO by the generated K_0.2_WO_3_ and NiWO_4_, thus contributing to the reduction in specific surface area of NiO. Furthermore, the pore size distribution shows that the aperture of mW/NiO is distributed over a smaller area compared with NiO. Figure S2 shows the CO_2_ physical adsorption capacities for the prepared samples. Compared with NiO, the adsorption capacities of mW/NiO heterojunction materials for CO_2_ decrease gradually with the increase in W content. These results indicate that pore blockage is the main reason for the reduction in specific surface area and the decrease in surface CO_2_ physical adsorption capacity, which is not the crucial factor determining the activity.

**Figure 3:**
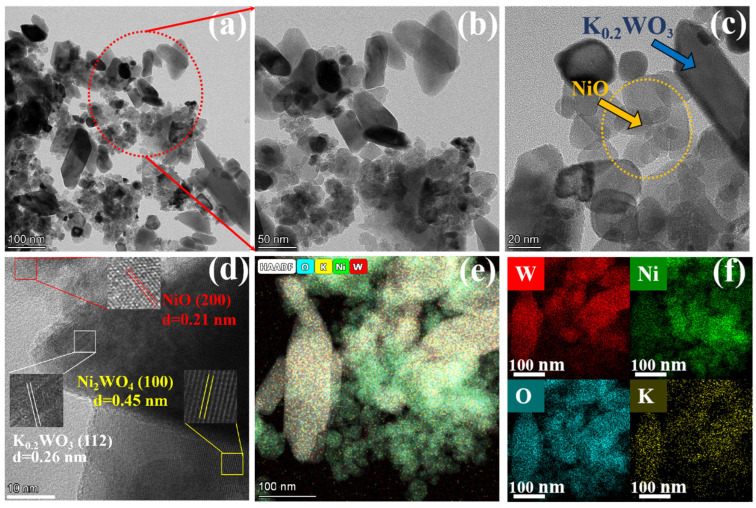
(**a**–**c**) TEM images; (**d**) HRTEM image; and (**e**,**f**) elemental mappings of 30W/NiO.

The microstructures and element distributions of NiO and 30W/NiO were studied by SEM and TEM, respectively. As presented in Figure S3a, the pristine NiO exhibits a uniform nanosphere, while it is changed by the constructed 30W/NiO S-scheme heterojunction. Furthermore, as displayed in Figure S3b, the nanosheet structure is found to be encapsulated in the agglomerated nanospheres, and the close contact of the heterogeneous structure may be favorable for the rapid transfer of charge and mass transfer of reactants on the surfaces. In addition, TEM morphological images under different magnifications are observed in Figure 3a,b, where it can be seen that 30W/NiO consists of irregularly shaped nanosheets and nanospheres. Based on our previous work, it can be inferred that the smaller nanosheets in Figure 3c can correspond to NiO, and while the sheet-like structure can be identified as K-tungsten bronze (K_0.2_WO_3_). Furthermore, the lattice spacing values of 0.21, 0.45, and 0.26 nm can be recognized in the high-resolution TEM image (Figure 3d), which correspond to the crystal planes of (200) of NiO, (100) of NiWO_4_, and (112) of K_0.2_WO_3_, respectively. The above results manifest the successful preparation of ternary heterojunction. In addition, the element distribution mappings (Figure 3e,f) show that O, K, Ni, and W are uniformly distributed, which illustrates that there is no obvious boundary among NiO, NiWO_4_, and K_0.2_WO_3_ ternary heterojunction.

**Figure 4:**
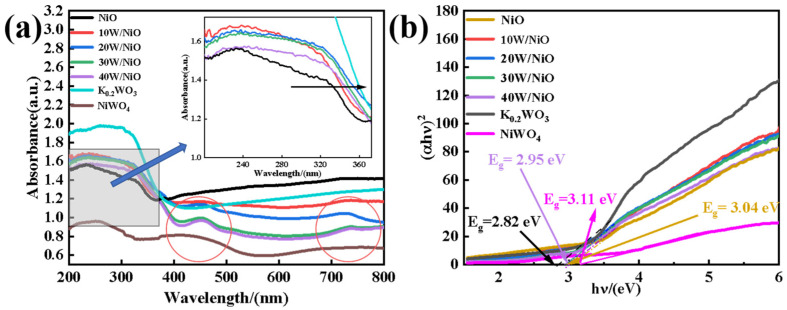
(**a**) Optical absorption properties (The circled part in the figure is the characteristic absorption peak of NiWO_4_, and the upper right corner is a local magnification of 200–370 nm); (**b**) corresponding (αhν)^2^-hν curves of NiO and mW/NiO.

Optical absorption is an important index to evaluate the property of a semiconductor in photocatalysis. UV-vis DRS in Figure 4a shows that the maximum absorptions of mW/NiO and NiO are mainly concentrated in the ultraviolet region, manifesting the basic characteristics of wide-gap semiconductors. By further observation, it is noteworthy that mW/NiO heterojunctions present a significant red-shift in optical absorption at the range of 200–370 nm, indicating that the optical absorption range of NiO can be broadened by the constructed heterojunction. In addition, the additional peaks of mW/NiO at 450 and 730 nm derive from the characteristic absorptions of NiWO_4_ [26]. Furthermore, Figure 4b displays that the band gaps of the mW/NiO samples are located at 2.95–3.04 eV, which is obviously narrower compared with pristine NiO. The above results demonstrate that the novel W/NiO heterostructure promotes the utilization of light compared with pure NiO [27]. Moreover, the band gaps of NiWO_4_ and K_0.2_WO_3_ are 3.11 eV and 2.82 eV, respectively.

**Figure 5:**
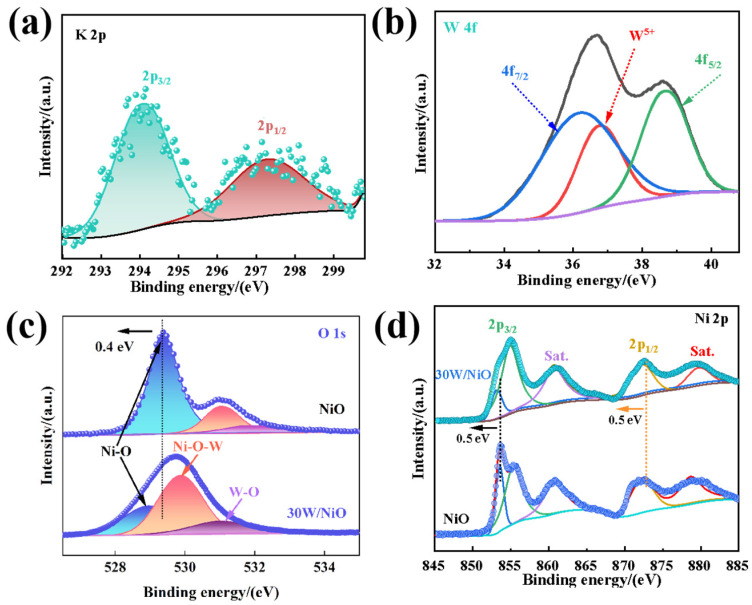
(**a**) K2p; (**b**) W4f; and (**c**) O1s and (**d**) Ni2p spectra of NiO and 30W/NiO.

The elemental compositions and chemical states of NiO and mW/NiO can be conveyed by XPS. It can be seen from Figure S4 that the characteristic peaks from the survey spectrum of 30W/NiO can be identified as Ni, O, W, and K, respectively, indicating that there is no element loss in the preparation process. It can be seen from Figure 5a that the double peaks of binding energies at 294.1 and 297.3 eV correspond to K 2p_3/2_ and K 2p_1/2_, showing that the K element exists in the form of K^+^ [28]. The fine scanning spectrum of the W element (Figure 5b) can be fitted into three peaks, with binding energies being located at 36.3, 36.9, and 38.7 eV, corresponding to W 4f_7/2_, W^5+^, and W 4f_5/2_, respectively [29,30], indicating that W exists in the mixed valence states of +5 and +6, which is evidence of the co-existence of K_0.2_WO_3_ and NiWO_4_. Different from NiO, the fitting peaks of O1s for 30W/NiO (Figure 5c) at binding energies of 528.9, 529.8, and 531.1 eV correspond to the lattice oxygen of NiO, NiWO_4_, and K_0.2_WO_3_, respectively [21,31]. Among them, the characteristic peak of Ni-O shows a 0.4 eV shift towards negative binding energy, indicating that the electron cloud density of oxygen on NiO decreases. The binding energies at 853.6 and 872.0 eV in Figure 5d are identified as Ni 2p_3/2_ and Ni 2p_1/2_ of NiO, respectively, manifesting the form of Ni^2+^ [21]. In addition, the bimodal peaks of binding energy near 860.6 and 870.7 eV correspond to the satellite peaks of Ni 2p. Further compared with NiO, the Ni 2p positions of 30W/NiO show a 0.5 eV shift towards the direction of decreasing binding energies, indicating that the electron cloud density on Ni sites increases, which can facilitate the reduction of CO_2_.

### 2.2. Photocatalytic CO_2_ Reduction Performance

**Figure 6:**
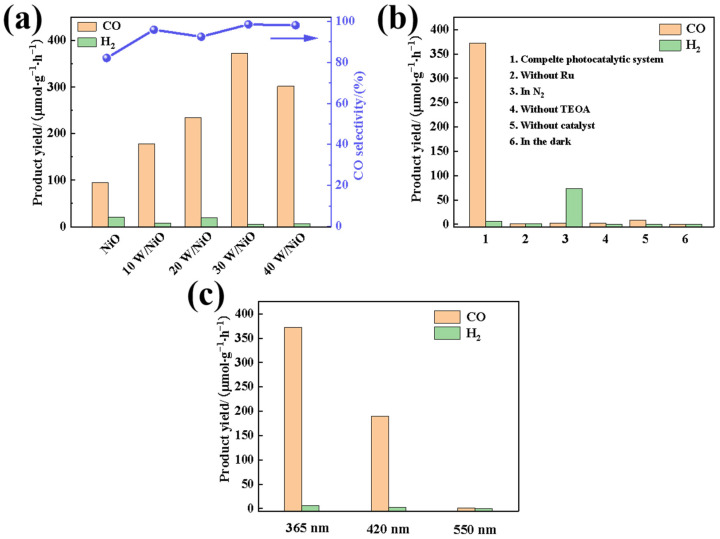
(**a**) Product yields and CO selectivities over NiO and mW/NiO under 365 nm irradiation; (**b**) product yields over 30W/NiO under different reaction conditions; (**c**) product yields of 30W/NiO at different wavelengths.

CO_2_ photoreduction tests were performed by using an LED lamp as the light source. There were no extra products found except CO and H_2_, and the yield unit was μmol·g^−1^·h^−1^. The product yields for all samples under the same conditions are shown in Figure 6a; the CO yield over NiO is 95 μmol·g^−1^·h^−1^, with a selectivity of 82%. Furthermore, the constructed heterojunction exhibits an excellent CO formation rate and high selectivity, where 30W/NiO shows a CO yield of 373 μmol·g^−1^·h^−1^, which is 3.9 times that of NiO. And its CO selectivity achieves 99%, which is 17% higher than that of NiO. It is noteworthy that the CO yield over mW/NiO enhances with the augment of the W element content and reach the maximum at 30%. These results indicate that the construction of the double S-scheme heterojunction promotes the formation and selectivity of CO. A further increase in the W element content at 40% induces the decrease yield of products, which may be due to the thickening of the NiWO_4_ layer coating and the disappearance of K_0.2_WO_3_ on the surface of NiO. In addition, to explore the influence of experimental conditions on the reactivity, a series of controlled trials were conducted. As shown in Figure 6b, in the absence of a photosensitizer, with TEOA or catalyst in the system, only trace products can be detected. In addition, only H_2_ can be detected when CO_2_ is replaced by N_2_, indicating that CO does not come from the decomposition of catalysts or sacrificial agents or organic solvents. In addition, no products were formed under dark conditions. The above results confirm that any change in conditions will have a great influence on product yield of the reaction system. As shown in Figure S5, the structure of the photocatalyst does not change before and after the reaction, which indicates that the prepared double S-scheme heterojunction is stable enough. To explore the properties at different wavelengths over the 30W/NiO sample, photocatalytic tests were also conducted under 360 nm, 420 nm, and 550 nm irradiation, respectively (Figure 6c). The CO yields of 30W/NiO are 373 μmol·g^−1^·h^−1^, 189.8 μmol·g^−1^·h^−1^, and 1.08 μmol·g^−1^·h^−1^ under the irradiation of 360 nm, 420 nm, and 550 nm, respectively. And Table S2 shows that the apparent quantum of 30W/NiO is 7.5 %, 4.2 %, and 0.1 % under 360 nm, 420 nm, and 550 nm wavelengths of light irradiation, respectively.

### 2.3. Discussion of Mechanism of Improving Photocatalytic Performance

**Figure 7:**
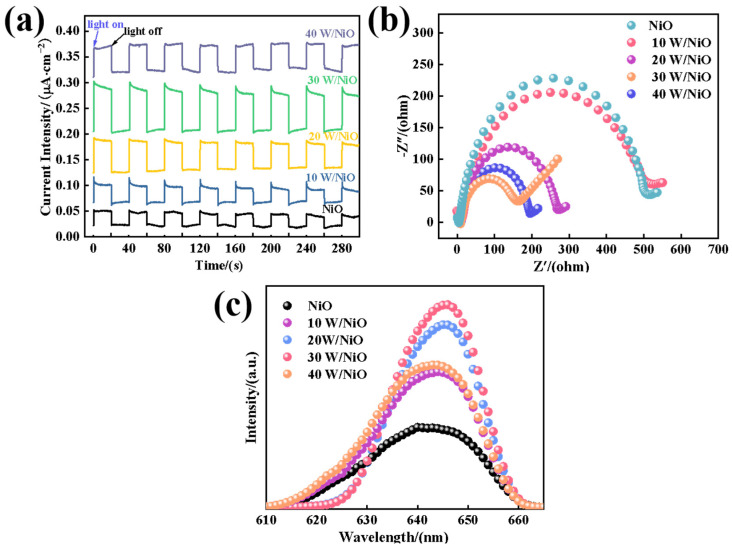
(**a**) Photocurrent response curves; (**b**) EIS; and (**c**) PL spectra for NiO and mW/NiO.

To study the separation efficiency of photogenerated charges, the transient current response curves were measured and are shown in Figure 7a. In the dark, the current response is quickly stabilized to a straight horizontal line, indicating that no additional current is generated without light. After the introduction of light, the current response significantly enhances, which is derived from the excited and transferred valence band electrons of the semiconductor under LED light irradiation, contributing to the additional current in the system. Compared with pristine NiO, mW/NiO exhibits obviously stronger current response under LED light irradiation, indicating the faster transfer of photoexcited charges. It is worth noting that if the proportion of the W element increases from 30% to 40%, the current response decreases instead, which may be due to the existence of a single S-scheme structure and the inhibited charge separation by excessive NiWO_4_. Furthermore, after the current suddenly increases when the light is turned on, it gradually decreases a bit, and then gradually increases a bit after the current suddenly decreases when the light is turned off. This may be attributed to the capture and release of photogenerated electrons by oxygen defects in the samples prepared by the molten salt method [32,33]. In addition, electrochemical impedance spectra (EIS) were adopted to explore the resistance in the process of charge transfer. As shown in Figure 7b, the arcs of mW/NiO show a smaller radius of curvature compared with bare NiO, indicating that the constructed double S-scheme heterojunction reduces the resistance for the reaction. These results suggest that the construction of heterojunction facilitates the transfer of photogenerated carriers, thus promoting the concentration of electrons involved in CO_2_ reduction.

Moreover, to further investigate the degree of electrons/holes recombination, PL spectra were collected to understand electron transfer for the prepared samples. Theoretically, the combination of electrons and holes can produce fluorescence, and generally, the lower the PL spectrum intensity is, the more efficient the separation of electrons and holes is. As shown in Figure 7c, at the excitation wavelength of 320 nm, the emission peaks of all samples at 645 nm were observed. In addition, the fluorescence intensity presents the order of 30W/NiO > 20W/NiO > 40W/NiO > 10W/NiO > NiO, indicating that the heterostructure emitted more obvious fluorescence. This might be attributed to the recombination of photogenerated carriers deep in the semiconductor bandgap due to high defects, as well as the recombination of photogenerated electrons in K_0.2_WO_3_ and NiWO_4_ with photogenerated holes in NiO in the double S-Scheme heterojunction 30W/NiO [33].

**Figure 8:**
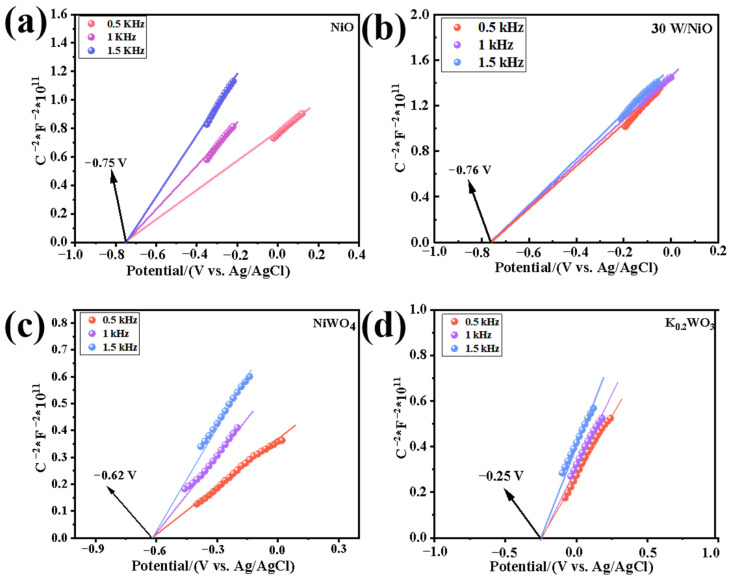
M-S plots of (**a**) NiO, (**b**) 30W/NiO, (**c**) NiWO_4_ and (**d**) K_0.2_WO_3_.

The investigation of semiconductor band structure is the premise of understanding the reaction mechanism. To study the flat band potential of NiO and 30W/NiO, a M-S test was employed. As shown in Figure 8, NiO, NiWO_4_, K_0.2_WO_3_, and 30W/NiO all correspond to n-type semiconductor properties with flat band potentials of −0.75 V, −0.62 V, −0.25 V, and −0.76 V (vs. Ag/AgCl, pH = 7), respectively. The flat band positions of NiO, NiWO_4_, K_0.2_WO_3_, and 30W/NiO are determined to be −0.55 V, −0.42 V, −0.05 V, and −0.56 V (vs. NHE, pH = 7), respectively, referring to previous work [34]. The conduction bands of NiO, NiWO_4_, K_0.2_WO_3_, and 30W/NiO are affirmed to be −0.65 V, −0.52 V, −0.15 V, and −0.66 V (vs. NHE, pH = 7), respectively. The coulomb repulsion between the electron-enriched conduction bands over K_0.2_WO_3_ and NiWO_4_ and the electron-enriched conduction band over NiO may be responsible for the conduction band to move upward. As a result, the flat and conduction band positions become more negative, which is thermodynamically more conducive to the output of electrons for CO_2_ reduction.

**Figure 9:**
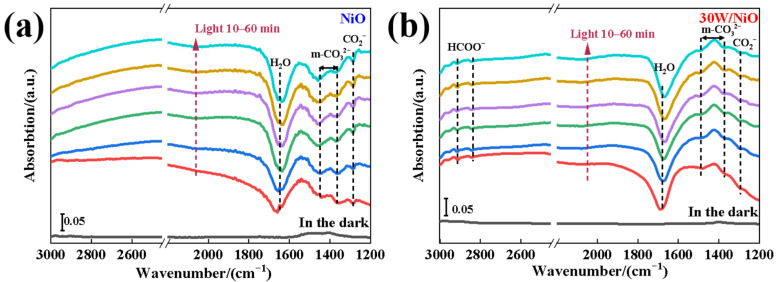
In situ FT-IR spectra of (**a**) NiO and (**b**) 30W/NiO.

To further understand the pathway and key intermediates during the reaction process, in situ FT-IR spectra of CO_2_ photoreduction on NiO and 30W/NiO were collected. Figure 9 presents the collected background curves after the saturation of H_2_O and CO_2_ adsorption in the dark (the black line) and the spectral curves every 5 min after illumination (the colored lines). The single peak at 1268 cm^−1^ is identified as the stretching vibration of the C-O bond of CO_2_^−^, while the double peaks at 2840 and 2910 cm^−1^ are ascribed to form HCOO^−^ [35,36]. In addition, the twin peaks near 1400 and 1495 cm^−1^ correspond to symmetric and asymmetric O-C-O stretching of m-CO_3_^2−^ [37]. In addition, the accumulated peak near 1647 cm^−1^ is attributed to the adsorption of H_2_O molecules. With the increase in illumination time, CO_2_^−^ and HCOO^−^ gradually accumulate on the surface of the catalyst, indicating that CO^2−^ and HCOO^−^ are important intermediates for the reduction of CO_2_ to CO [38,39]. Compared with the pristine NiO, the in situ FT-IR spectra of 30W/NiO present additional cumulative peaks of HCOO^−^ at 2840 and 2910 cm^−1^ [40], indicating that HCOO^−^ plays a key role in promoting the conversion of CO_2_ to CO during the whole reaction. Furthermore, it can be seen from the figure that the accumulation of m-CO_3_^2−^ is slower on 30W/NiO than that of NiO, demonstrating that the construction of the heterojunction reduces the generation of by-products and promotes the highly selective generation of CO.

**Figure 10:**
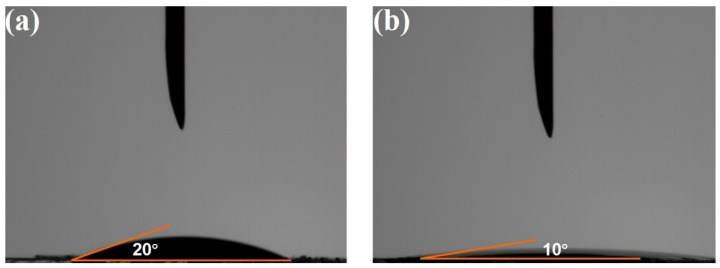
Contact angles with water over (**a**) NiO and (**b**) 30W/NiO.

To investigate the abilities of the adsorbed reactants to obtain protons on the surface of the samples, the contact angles with water over NiO and 30W/NiO at room temperature were measured, as shown Figure 10. The contact angle between NiO and water is 20°, while the corresponding contact angle over 30W/NiO is minimized to 10°. It indicates that the construction of the double S-scheme heterostructure promotes the dispersion of H_2_O molecules on the material’s surface. Moreover, the wettable surface hydrophilic layer can facilitate the adsorption of CO_2_ molecules to protons, thus contributing to the hydrogenation reduction ability of CO_2_ (HCOO^−^), which is consistent with the enhanced hydroxyl adsorption from the FT-IR results.

**Figure 11:**
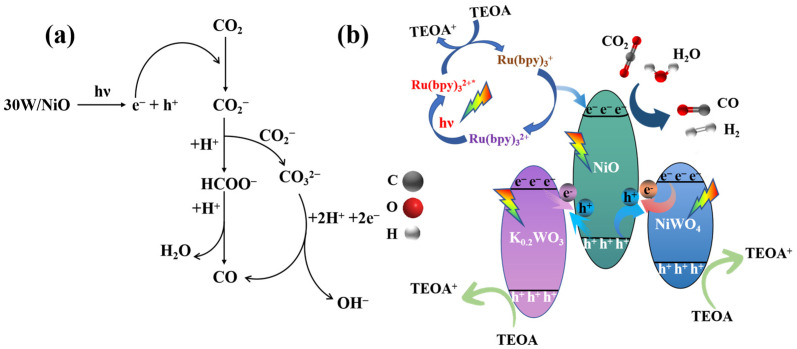
(**a**) The transformation process diagram and (**b**) the possible reaction mechanism of CO_2_ photoreduction over mW/NiO.

Combined with the discussion of the above experimental results, to better understand the whole reaction process, the possible paths and the mechanism of CO_2_ photoreduction are proposed (Figure 11a). The transformation diagram of intermediate products can be confirmed from the discussion of in situ FT-IR results, in which CO_2_ is converted into CO on mW/NiO through the following process: (i) CO_2_ combines with electrons from the conduction band to form CO_2_^−^. (ii) A part of CO_2_^−^ obtains the protons to further form HCOO^−^, while the other part self-combines to form carbonate CO_3_^2−^. (iii) HCOO^−^ further obtains the protons and removes a molecule of water to form CO. Meanwhile, CO_3_^2−^ with the formation of by-products also acquires protons and electrons to form CO.

The band gap structure of the samples was obtained by UV-visible spectroscopy and a M-S test. The band gap of NiO is 3.04 eV, and its conduction band and valence band are −0.65 V and 2.39 V, respectively. The band gap of NiWO_4_ is 3.11 eV, and its conduction band and valence band are −0.52 V and 2.59 V, respectively. The band gap width of K_0.2_WO_3_ is 2.82 eV, and its conduction band and valence band positions are −0.15 V and 2.67 V, respectively. Therefore, the staggered band positions between NiO and NiWO_4_, NiO and K_0.2_WO_3_ conform to the S-type heterojunction structure. In addition, the work function and Fermi level of NiO are about 4.6 eV and 0.02 V [41], respectively, and the work function and Fermi level of NiWO_4_ are about 5.61 eV and −0.5 V [42], respectively. In fact, K_0.2_WO_3_ is K-doped WO_3_, so the work function and Fermi level of K_0.2_WO_3_ are similar to WO_3_. Therefore, the work function and Fermi level of K_0.2_WO_3_ are about 6.88 eV and −3.42 V [43], respectively. Therefore, when NiO with a higher Fermi level and a smaller work function is in close contact with K_0.2_WO_3_, electrons will flow from NiO to K_0.2_WO_3_ until Fermi energy reaches equilibrium [44]. Therefore, a built-in electric field from NiO to K_0.2_WO_3_ is formed at the interface between NiO and K_0.2_WO_3_. Similarly, a built-in electric field from NiO to NiWO_4_ is also formed between NiO and NiWO_4_. Therefore, when NiO, NiWO_4_, and K_0.2_WO_3_ absorb photon energy to excite photogenerated carriers, the photogenerated electrons of NiWO_4_ and K_0.2_WO_3_ will flow to NiO and recombine with the photogenerated holes of NiO under the synergistic effect of the built-in electric field and band bending, thus promoting the separation of photogenerated carriers and improving its photocatalytic CO_2_ reduction performance. Therefore, the possible mechanism is presented in Figure 11b. Under exposure to the light, the semiconductors are simultaneously excited, and each generated electron/hole pairs (e^−^-h^+^). Subsequently, the excited electrons on CB of K_0.2_WO_3_ and NiWO_4_ combined with the holes on VB of NiO and eventually quenched. Furthermore, the holes accumulated on VB of K_0.2_WO_3_ and NiWO_4_ are consumed by TEOA, while TEOA is oxidized to diethanolamine and glycolaldehyde [45]. At the same time, Ru(bpy)_3_^2+^ is activated to excited Ru(bpy)_3_^2+*^ under light and then quenched by TEOA reduction to form Ru(bpy)_3_^+^. Subsequently, e^−^ from Ru(bpy)_3_^+^ migrate to the CB of NiO and accumulate, and Ru(bpy)_3_^+^ is oxidized to Ru(bpy)_3_^2+^. The enriched excited e^−^ in CB of NiO are further accepted by the adsorbed CO_2_ molecules and combine with the protons from water to form HCOO^−^. HCOO^−^ further obtains the protons and removes a molecule of water, and eventually converts into the product CO.

## 3. Experimental Section

### 3.1. Materials

The nickel nitrate hexahydrate (Ni(NO_3_)_2_∙6H_2_O, Sinopharm Chemical Reagents Co., Ltd., Shanghai, China), sodium hydroxide (NaOH, Shanghai Aladdin Biochemical Technology Co., Ltd., Shanghai, China), anhydrous lithium chloride (LiCl, Shanghai Maclin Biochemical Technology Co., Ltd., Shanghai, China), potassium chloride (KCl, Xilong Scientific Co., Ltd., Shantou, China), tungstic acid (H_2_WO_4_ Sinopharm Chemical Reagent Co., Ltd., Shanghai, China), anhydrous ethanol (CH_3_CH_2_OH, Xilong Scientific Co., Ltd., Shantou, China), acetonitrile (MeCN, Xilong Scientific Co., Ltd., Shantou, China), triethanolamine (TEOA, Xilong Scientific Co., Ltd., Shantou, China), and [Ru(bpy)_3_]Cl_2_ ∙6H_2_O (Shanghai McLean Biochemical Technology Co., Ltd., Shanghai, China) chemicals used in this work are analytically pure and commercially available without further purification. The used gases (99.99% N_2_, 99.99% CO_2_, Ganzhou Jianli Gas Co., Ltd., Ganzhou, China) were purchased from the supplier and used directly upon receipt.

### 3.2. Synthesis of Precursor Ni(OH)_2_

Ni(OH)_2_ was synthesized as follows: 10 mmol Ni(NO_3_)_2_∙6H_2_O was dissolved in 40 mL deionized (DI) water under magnetic agitation, and 20 mmol NaOH was added and stirred for 30 min. After filtration, the precipitation was collected and washed with 10 mL DI water and anhydrous ethanol, respectively. The obtained Ni(OH)_2_ was dried at 60 °C for 8 h.

### 3.3. Synthesis of NiO

A total of 5 mmol Ni(OH)_2_, 2.7 g LiCl, and 3.3 g KCl were fully ground and transferred to an alumina crucible and calcined at 400 °C for 3 h. The reacted block was fully dissolved in appropriate DI water and filtered and washed with DI water and anhydrous ethanol several times alternately. The obtained solid was dried at 60 °C for 8 h, and NiO was successfully prepared.

### 3.4. Synthesis of K_0.2_WO_3_/NiO/NiWO_4_ Heterojunction

The preparation of NiWO_4_/NiO/K_0.2_WO_3_ heterojunction was based on 2.3, except that a different fraction of H_2_WO_4_ was introduced to the synthesis process of NiO. Ensuring that the molar ratio of W/Ni = 10%, 20%, 30%, and 40% on the dosages of H_2_WO_4_ and NiO, the as-synthesized photocatalysts were denoted as mW/NiO (m = 10, 20, 30, and 40). In addition, NiWO_4_ can be obtained when W/Ni = 1.

### 3.5. Synthesis of K_0.2_WO_3_

A total of 0.2 g H_2_WO_4_, 2.7 g LiCl, and 3.3 g of KCl were thoroughly ground and transferred to an alumina crucible, then calcined at 400 °C for 3 h. The reacted block was dissolved in appropriate DI water and filtered and washed alternately with DI water and anhydrous ethanol several times. The obtained solid was dried at 60 °C for 8 h, and K_0.2_WO_3_ was successfully prepared.

### 3.6. Photocatalytic CO_2_ Reduction

The photocatalytic CO_2_ photoreduction reactions were carried out under liquid–solid condition and evaluated under an 80 W LED lamp (Zhenjiang Yinzhu Chemical Technology Co., Ltd., Zhenjiang, China); the illumination wavelength was 365 nm, 420 nm, and 550 nm, respectively. Generally, 30 mg catalyst, 5 mg [Ru(bpy)_3_]Cl_2_·6H_2_O (denote as Ru), 3 mL acetonitrile (MeCN), 2 mL DI water, and 1 mL triethanolamine (TEOA) were added to the quartz reactor with a volume of 50 mL. Before the reaction began, the reactor was exhausted using pure CO_2_ in the dark for 30 min. After exposure to the light, 1 mL product gas was detected by gas chromatographic (GC 9790 II, FuLi, Taizhou, China) equipped with both TCD and FID at 2 h. CO selectivity was calculated using the following formula:

(1) $${\mathrm{CO}\;\mathrm{Selectivity}\;(S}_{\mathrm{CO}})=\frac{Y_{\mathrm{CO}}}{Y_{\mathrm{CO}}{+Y}_{H_{2}}}\times 100\%$$

where $Y_{CO}$ and $Y_{H_{2}}$ represent the yield of CO and H_2_, respectively.

Furthermore, the optical powers at different wavelengths were measured via an optical power meter, with a probe area of 1 × 1 cm^2^ to contact light. The light irradiation area is 2.5 × 2.5 cm^2^, and apparent quantum efficiency (AQE) was calculated using the following formula:

(2) $$AQE=\frac{2\times the\;number\;of\;evolved\;CO\;molecules\;}{N}\times 100\%$$

(3) $$N=\frac{E\lambda }{hc}\;$$

N: the number of incident photons;
E: the accumulated light energy in the given area (J);
λ: the wavelength of the light;
h: the Plank constant (6.626 × 10^−34^ J·s);
c: the velocity of light (3 × 10^8^ m·s^−1^).

### 3.7. Characterizations

XRD patterns for prepared materials were characterized by Bruker D8 advanced powder X-ray diffractometer (Bruker company, Munich, Germany). Fourier transform infrared spectra (FTIR) were collected by the Nicolet 5700 FT-IR spectrometer using KBr pellets as reference (Nicolet Instrument Co., U.S.A, Madison, WI, USA). Nitrogen adsorption–desorption isotherms and CO_2_ adsorption capacities for samples were analyzed using ASAP 2020 automatic analyzer (Micromeritics Instrument Corporation, Norcross, GA, USA). The samples were degassed at 150 °C for two hours before analysis. The microstructures of the synthesized specimens were investigated using field emission scanning electron microscopy (SEM, Zeiss Sigma 500, Carl Zeiss AG, Jena, Germany) and transmission electron microscopy (TEM, Jeol JEM-2100F, Nippon Electric Company, Akishima, Japan). Ultraviolet-visible diffuse reflectance spectra (UV-Vis DRS) were collected using a UV-vis spectrophotometer (UV-2600, Shimadzu, Unocal Corporation, El Segundo, CA, USA). XPS measurements were adopted by X-ray photoelectron spectrometer (XPS, Thermo Fisher K-Alpha, Thermo Fisher Scientific Inc., Waltham, MA, USA). The photoluminescence and fluorescence lifetime spectra were performed by FLS980 fluorescence spectrometer (Edinburgh Instruments, Livingston, UK) at the excitation wavelength of *λ* = 300 nm. In situ diffused reflection FTIR spectra were collected using a Bruker TENSOR II spectrometer (Bruker company, Germany). Then, 30 mg of photocatalyst was placed in the infrared pool, cooled by liquid nitrogen, and vacuumized. Humid CO_2_ gas was added into the infrared pool to realize the adsorption/desorption balance and then time-resolved FTIR spectra from 1200 to 3000 cm^−1^ were collected on LED lamp irradiation. Photoelectrochemical tests were performed in a three-electrode system using an electrochemical workstation (CHI660E, CH Instruments Inc., Bee Cave, TX, USA), with 0.1 M Na_2_SO_4_ as the electrolyte, the photocatalytic material on FTO as the working electrode (catalyst distribution area: 0.25 cm^2^), the saturated Ag/AgCl and platinum electrode as the reference electrode and counter electrode, respectively, and an 80 W LED lamp as the light source. Furthermore, the semiconductor type and flat band potential for specimens were determined by Mott–Schottky (M–S) tests.

## 4. Conclusions

In this work, K_0.2_WO_3_/NiO/NiWO_4_ ternary double S-scheme heterojunction was synthesized via a molten salt method by introducing tungstic acid in the preparation process of NiO. This heterojunction exhibited redshifted optical absorption compared to pristine NiO, enhancing light utilization. The heterojunction enhanced the charge separation and transfer of NiO, thereby minimizing the mass transfer resistance in the reaction process. In addition, the in situ FT-IR test revealed that the construction of heterojunction promoted the formation of HCOO− key intermediates in the CO_2_ reduction process, and reduced the formation of by-products, contributing to the improved CO_2_ photoreduction performance with a the high apparent quantum efficiency (7.5%) under 365 nm LED irradiation.

## Data Availability

Data are contained within the article and Supplementary Materials.

## Supplementary Materials

The following supporting information can be downloaded at: https://www.mdpi.com/article/10.3390/molecules30081804/s1, Figure S1: N_2_ adsorption-desorption curve; Figure S2: CO_2_ adsorption curve for NiO and mW/NiO; Figure S3: SEM images of prepared samples: (a) NiO; (b) 30W/NiO; Figure S4: XPS survey spectra of 30W/NiO; Figure S5: XRD patterns of 30W/NiO before and after reaction; Table S1: BET parameters for NiO and mW/NiO; Table S2: Optical power, CO yield and AQEs over 30W/NiO under 2 h illumination at different wavelengths.
