# Supplementary material for: One-Step Molten Salt Constructing Double S-Scheme K0.2WO3/NiO/NiWO4 Heterojunction for Photocatalytic CO2 Reduction
Source: Molecules. 2025 Apr 17;30(8):1804. doi: 10.3390/molecules30081804 (PMC12029344; doi:10.3390/molecules30081804)
Supplement: Supplementary file 1 [file molecules-30-01804-s001.zip › molecules-3560308-supplementary.pdf]

Supporting Information

One-step molten salt constructing a novel dual S-scheme $K_{0.2}WO_3/NiO/NiWO_4$ heterojunction for photocatalytic reduction of CO_2

Wentao Xiang,[⊥a] Zhenzhen Yu,[⊥a] Fulin Wang,[a] Chensheng Zhou,[a] Kangqiang Lu,[a] Weiya Huang,[a] Changlin Yu,[c] Man Zhou,[*b] Kai Yang[*a]

a, School of Chemistry and Chemical Engineering, Jiangxi Provincial Key Laboratory of Functional Crystalline Materials Chemistry, Jiangxi University of Science and Technology, Ganzhou 341000, Jiangxi, China.

b, School of Pharmaceutical Sciences, Gannan Medical University, Ganzhou 341000, Jiangxi, China.

c, School of Chemical Engineering, Guangdong University of Petrochemical Technology, Maoming 525000, Guangdong, China

1. *Corresponding author: Kai Yang
   E-mail: yangkai@jxust.edu.cn
2. *Corresponding author: Man Zhou
   E-mail: baiyuwawa-zhouman@163.com

Wentao Xiang and Yu Zhenzhen contributed equally to this work and could be considered as co-first authors.

1. Experimental

1.1. Materials

The nickel nitrate hexahydrate ($Ni(NO_3)_2 \cdot 6H_2O$, Sinopharm Chemical Reagents Co., Ltd.), sodium hydroxide (NaOH, Shanghai Aladdin Biochemical Technology Co., Ltd.), anhydrous lithium chloride (LiCl, Shanghai Maclin Biochemical Technology Co., Ltd.), potassium chloride (KCl, Xilong Scientific Co., Ltd.), tungstic acid (H_2WO_4 Sinopharm Chemical Reagent Co. Ltd.), anhydrous ethanol (CH_3CH_2OH, Xilong Scientific Co., Ltd.), acetonitrile (MeCN, Xilong Scientific Co., Ltd.), triethanolamine (TEOA, Xilong Scientific Co., Ltd.) and $[Ru(bpy)_3]Cl_2 \cdot 6H_2O$ (Shanghai McLean Biochemical Technology Co., Ltd.) chemicals used in this work are analytically pure and commercially available without further purification. The used gases (99.99% N_2, 99.99% CO_2) were purchased from the supplier and used directly upon receipt.

1.2. Synthesis of $Ni(OH)_2$ precursor

$Ni(OH)_2$ was synthesized as follows: 10 mmol $Ni(NO_3)_2 \cdot 6H_2O$ was dissolved in 40 mL deionized (DI) water under magnetic agitation, and 20 mmol NaOH were added and stirred for 30 min. After filtration, the precipitation was collected and washed with 10 mL DI water and anhydrous ethanol, respectively. The obtained $Ni(OH)_2$ was dried at 60°C for 8 h.

1.3. Synthesis of NiO photocatalyst

5 mmol $Ni(OH)_2$, 2.7 g LiCl and 3.3 g KCl were fully ground and transferred to an alumina crucible, and calcined at 400 °C for 3 h. The reacted block was fully dissolved in appropriate DI water and filtered and washed with DI water and anhydrous ethanol for several times alternately. The obtained solid was dried at 60°C for 8 h, NiO was successfully prepared.

1.4. Synthesis of $NiWO_4/NiO/K_{0.2}WO_3$ heterojunction

The preparation of $NiWO_4/NiO/K_{0.2}WO_3$ heterojunction was based on 2.3, except that different fraction of H_2WO_4 was introduced to the synthesis process of NiO. Ensuring that the molar

ratio of W/Ni= 10 %, 20 %, 30 % and 40 % on the dosages of H_2WO_4 and NiO, the as-synthesized photocatalysts were denoted as mW/NiO (m= 10, 20, 30 and 40).

1.5. Characterizations

XRD patterns for prepared materials were characterized by Bruker D8 advanced powder X-ray diffractometer. Fourier transform infrared spectra (FTIR) were collected by the Nicolet 5700 FT-IR spectrometer using KBr pellets as reference. Nitrogen adsorption-desorption isotherms and CO_2 adsorption capacities for samples were analyzed using ASAP 2020 automatic analyzer. Samples were degassed at 150 °C for two hours before analysis. The microstructures of the synthesized specimens were investigated using field emission scanning electron microscopy (SEM, Zeiss Sigma 500) and transmission electron microscopy (TEM, Jeol JEM-2100F). Ultraviolet-visible diffuse reflectance spectra (UV-Vis DRS) were collected using a UV-vis spectrophotometer (UV-2600, Shimadzu). XPS measurements were adopted by X-ray photoelectron spectrometer (XPS, Thermo Fisher K-Alpha). The photoluminescence and fluorescence lifetime spectra were performed by FLS980 fluorescence spectrometer at the excitation wavelength of λ=300 nm. In-situ diffused reflection FTIR spectra were collected using a Bruker TENSOR II spectrometer. 30 mg of photocatalyst was placed in the infrared pool, cooled by liquid nitrogen and vacuumized. Humid CO_2 gas was added into the infrared pool to realize the adsorption/desorption balance and then time-resolved FTIR spectra from 1200 to 3000 cm^{-1} were collected on LED lamp irradiation.

1.6. Photoelectrochemical tests

Photoelectrochemical tests were performed in a three-electrode system using electrochemical workstation (CHI660E, China), with 0.1 M Na_2SO_4 as the electrolyte, the photocatalytic material on FTO as the working electrode (catalyst distribution area: 0.25 cm^2), the saturated Ag/AgCl and platinum electrode as reference electrode and counter electrode, respectively, and 80 W LED lamp as light source. Furthermore, the semiconductor type and flat band potential for specimens were

determined by mott-schottky (M–S) tests.

1.7. Photocatalytic CO_2 reduction test

The photocatalytic CO_2 photoreduction reactions were carried out under liquid-solid condition and evaluated under 80 W LED lamp (zhenjiang Yinzhu Chemical Technology Co., LTD), the illumination wavelength was 365 nm, 420 nm and 550 nm, respectively. Generally, 30 mg catalyst, 5 mg $[Ru(bpy)_3]Cl_2\cdot 6H_2O$ (denote as Ru), 3 mL acetonitrile (MeCN), 2 mL DI water and 1 mL triethanolamine (TEOA) were added to the quartz reactor with a volume of 50 mL. Before the reaction began, the reactor was exhausted using pure CO_2 in the dark for 30 min. After exposure to the light, 1 mL product gas was detected by gas chromatographic (GC 9790 II, FuLi) equipped with both TCD and FID at 2 h.

1.8. Calculation of CO selectivity and apparent quantum efficiency (AQE)

CO selectivity was calculated using the following formula:

$$\text{CO Selectivity } (S_{\text{CO}}) = \frac{Y_{\text{CO}}}{Y_{\text{CO}} + Y_{\text{H}_2}} \times 100\%$$

Where Y_{CO} and Y_{H2} represent the yield of CO and H_2, respectively.

Furthermore, the optical powers at different wavelengths were measured via an optical power meter, with the probe area of 1×1 cm^2 by contacting light. The light irradiation area is 2.5×2.5 cm^2, AQE was calculated using the following formula:

$$AQE = \frac{2\times \text{the number of evolved CO molecules}}{N} \times 100\%$$

$$N = \frac{E\lambda}{hc}$$

N: the number of incident photons;

E: the accumulated light energy in the given area (*J*);

λ: the wavelength of the light;

h: the Plank constant (6.626×10^{-34} *J•s*)

c: the velocity of light (3×10^{8} $m{\bullet}s^{-1}$)

2. Supporting Figures

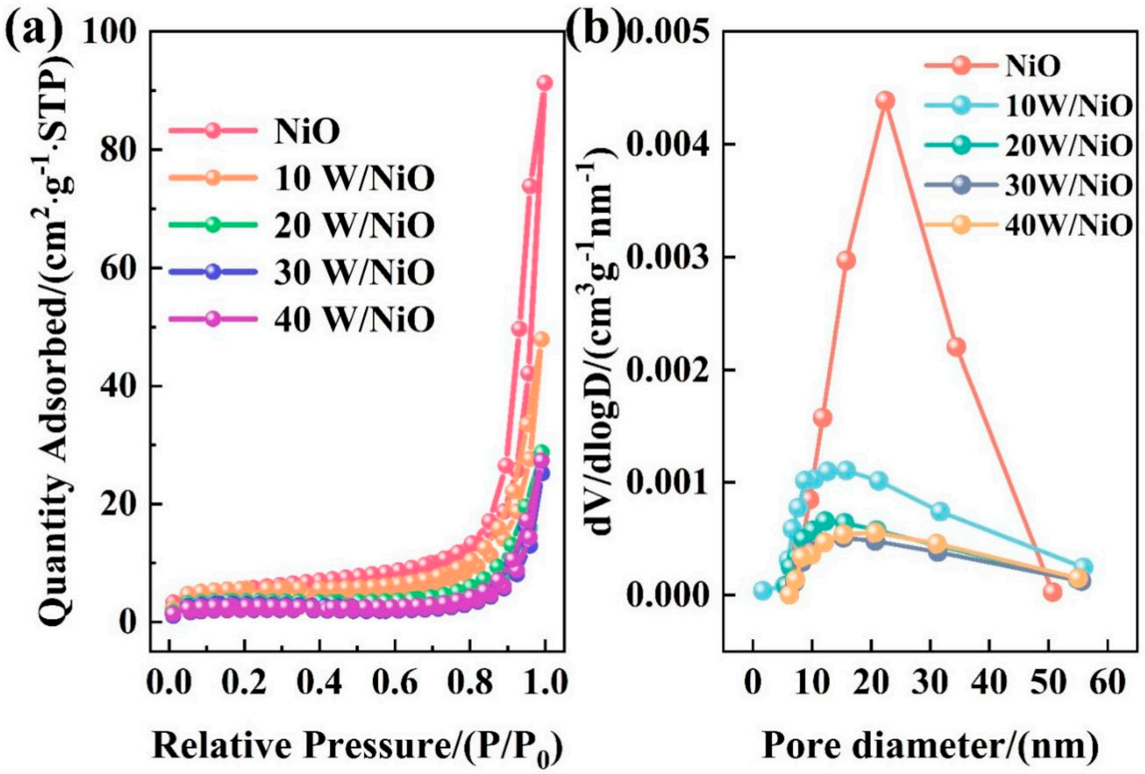

Figure S1 (a) N_2 adsorption-desorption curve and (b) pore size distribution of NiO and mW/NiO.

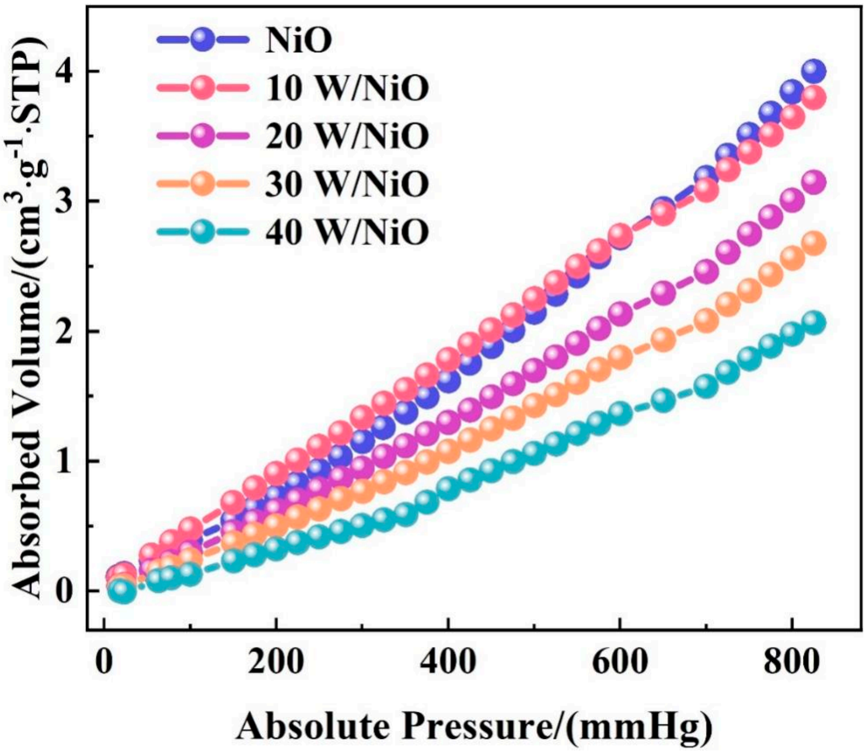

Figure S2 CO_2 adsorption curve for NiO and mW/NiO.

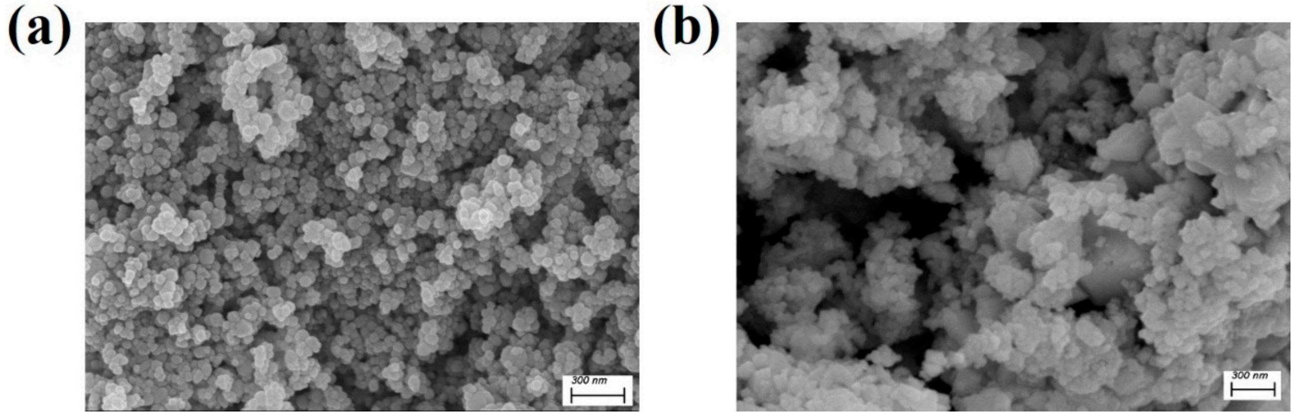

Figure S3 SEM images of prepared samples: (a) NiO; (b) 30W/NiO.

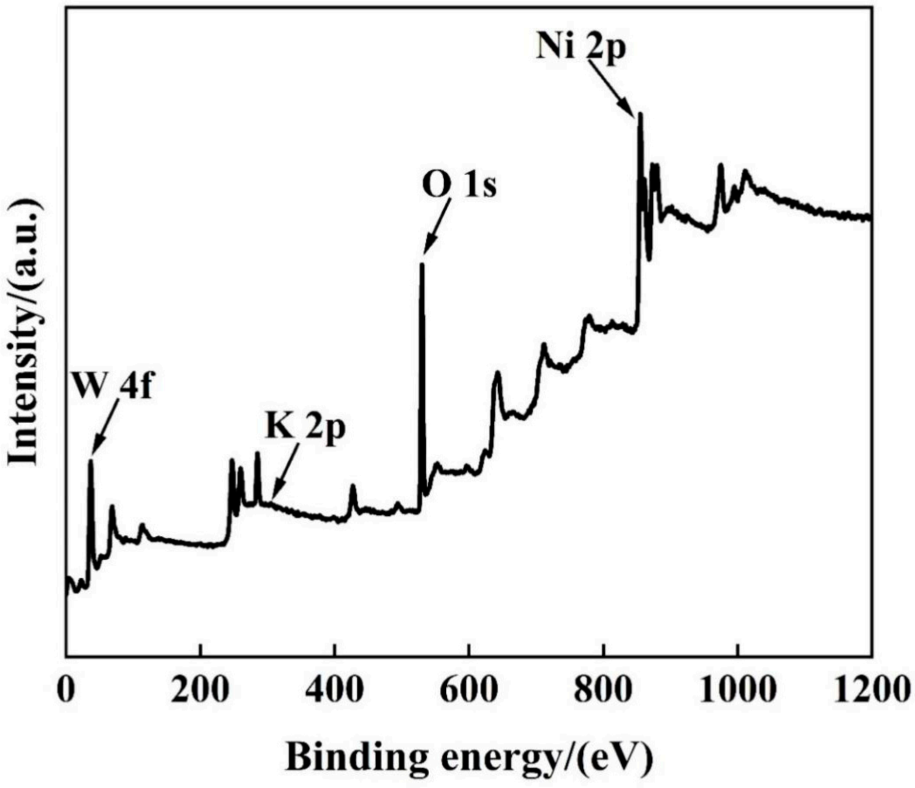

Figure S4 XPS survey spectra of 30W/NiO

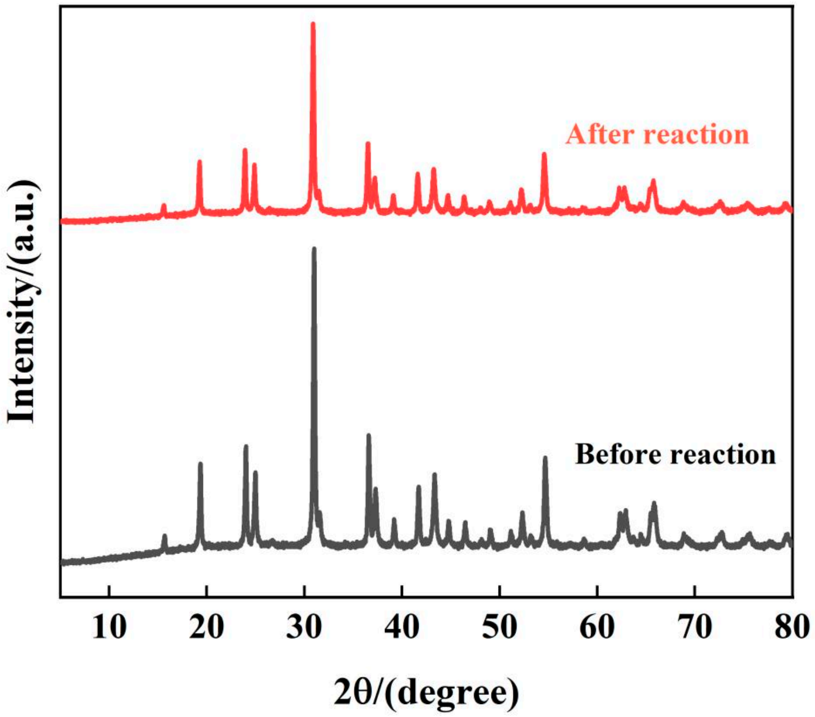

Figure S5 XRD patterns of 30W/NiO before and after reaction.

Table S1. BET parameters for NiO and mW/NiO.

Sample	BET surface area (m^2/g)	Pore volume (cm^3/g)	Pore size (nm)
NiO	19.14	0.14	29.51
10W/NiO	14.05	0.074	21.10
20W/NiO	8.47	0.044	20.94
30W/NiO	6.14	0.039	25.13
40W/NiO	6.62	0.042	25.50

Table S2. Optical power, CO yield and AQEs over 30W/NiO under 2 h illumination at different wavelengths.

**Wavelength/(*nm*)**	**Optical power/(*mW*)**	**CO yield/(μ*mol*·g^{-1}·h^{-1})**	**AQE/(%)**
365	456.4	373	7.5
420	358.7	189.8	4.2
550	59.2	1.08	0.1
